# X-RAY EMISSION FROM MATERIALS PROCESSING LASERS

**DOI:** 10.1093/rpd/ncy126

**Published:** 2018-09-05

**Authors:** R Behrens, B Pullner, M Reginatto

**Affiliations:** Physikalisch-Technische Bundesanstalt, Bundesallee 100, D-38116 Braunschweig, Germany

## Abstract

The emission of laser induced X-rays from materials processing ultra-short pulsed laser systems was measured. The absolute spectral photon fluence was determined using a thermoluminescence detector based few-channel spectrometer. The spectra at 10 cm from the laser focus were in the energy region between 2 and 25 keV with mean energies of ~4–6 keV (when weighted by fluence or directional dose equivalent) and up to 13 keV (when weighted by ambient dose equivalent). The operational quantities, $\overset{\cdot }{H}$′(0.07), $\overset{\cdot }{H}$′(3) and $\overset{\cdot }{H}$*(10), were determined to be in the order of 1600–7300, 16–71 and 1–4 mSv per hour processing time, respectively, depending on the material and condition of the workpiece. The dose contribution due to photons above 30 keV was for all quantities negligible, i.e. below 10^−3^.

## INTRODUCTION

Ultra-short pulsed lasers have been developed in the last decades for both research and various applications^(1)^. In some of these applications, the main purpose is the production of ionizing radiation, with laser intensities at the focus of the order of up to 10^22^ W/cm^2^. Much lower intensities have been in use for materials processing without the production of ionizing radiation^(2)^. However, in the last few years, larger intensities of up to the order of 10^14^ W/cm^2^ are in use for materials processing resulting in an unwanted production of ionizing radiation^(3, 4)^. Advantages of such systems are, for example, extremely small holes (a few tens of μm in diameter) with nearly perfect edges due to the evaporation of the material, as opposed to holes produced by melting the material. The evaporation creates a plasma which by nature contains ions. The electrons from the plasma produce bremsstrahlung and characteristic X-rays. These photons are expected to be in the energy region of a few tens of keV, as the laser reaches intensities in the order of 10^14^ W/cm^2^^(5)^. Due to the ultra-short laser pulses, which are in the order of less than a picosecond, the photons are correspondingly pulsed and cannot be measured using an active counting method like a Germanium spectrometer due to pile-up effects. To reduce the photon fluence, and consequently the pile-up effects, an active spectrometer could be positioned at larger distances, but this would result in an unwanted attenuation especially for low energy photons.

In this work, the unwanted emission of ionizing radiation is investigated for a materials processing laser system which is ready for routine production.

## EXPERIMENTS

### Laser

The laser system GL5 evo, from the company GFH GmbH^(6)^, was used for the experiments. Its parameters are listed in Table 1. The field of applications ranges from ablation and coating removal processes to engravings and micro drilling with high aspect ratios.

**Table 1.** Laser parameters.

Characteristic	Value
Wavelength	1030 nm
Average power	78 W
Pulse energy	195 μJ
Pulse length (FWHM)^a^	924 fS
Repetition rate	400 kHz
Focus diameter	16 μm
Focus intensity	2.1·10^14^ W/cm^2^

^a^Full width at half maximum.

### Measuring device

#### Few-channel spectrometer

**Figure 1.**
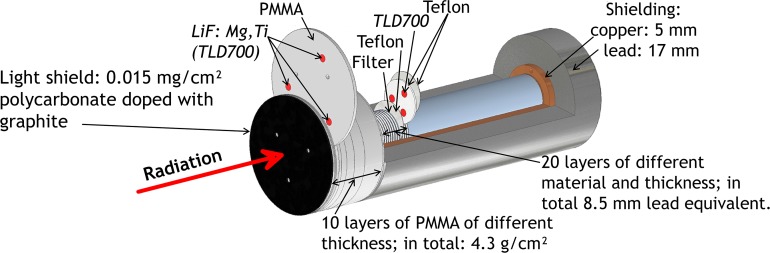
Sketch of the few-channel spectrometer.

**Figure A1.**
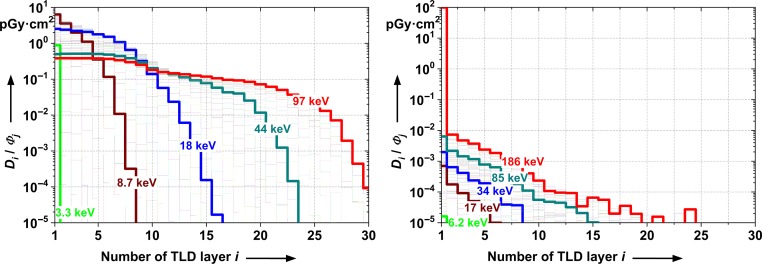
Response functions (depth dose curves) for mono-energetic photons (left) and electrons (right): dose per incident fluence at the front of the spectrometer vs. TLD layer in the spectrometer, calculated for 11 cm from a point. From one line to the next, the energy differs by a factor of 1.0674. For a few energy channels the mean energy is given. Note that both scales are logarithmic.

A thermoluminescence detector (TLD) based few-channel spectrometer^(7)^ was used to measure the particle spectrum of the emitted radiation (Figure 1). To determine the incident spectrum, it is necessary to know its response functions, i.e. the dose in the 30 TLD layers per incident fluence for mono-energetic particles, which have been calculated for photons and electrons. From this and the measured doses in the TLDs the absolute fluence spectrum can be determined by means of deconvolution. (The TLDs were calibrated outside of the spectrometers in a ^137^Cs reference radiation field of PTB traceable to PTB’s primary standard for dose measurements.) It is suitable for photons and electrons (keV range up to the MeV range). In the past, it has been used for both laser induced emissions at laser intensities of up to 10^19^ W/cm^2^^(8, 9)^ and in pure photon fields^(10)^ in the energy range from several keV to up to several MeV. As the laser intensity in this application is much lower, in the order of 10^14^ W/cm^2^ (Table 1), particles from a few keV to up to some 10 keV are expected. Therefore, the filter thicknesses were reduced (see Table A1 in Appendix 1) as compared to the earlier version^(7)^. The response functions for this new version were calculated using EGS4 (EGS4 was used as the original version of the spectrometer dates to 1998. At that time EGS4 was the up to date version of the code.) with low energy extensions^(11–13)^ for photons from 2 to up to 100 keV and for electrons from 4 to up to 192 keV (see Figure A1 in Appendix 1). For electrons, slightly larger energies were chosen as they are the primary ionizing particles produced in the laser plasma and, consequently have, on average, larger energies than the secondary photons produced via the bremsstrahlung process.

#### Scintillator dosemeter

In order to determine the effective irradiation time during the experiments, a scintillation dosemeter for ambient dose equivalent, H*(10), from the company Automess^(14)^, was used as radiation monitor. The background dose rate was determined to be 0.052 ± 0.007 μSv/h (standard deviation of the single values). The duration of the irradiation was selected by choosing the time for which the indication of the dosemeter exceeded the background dose rate plus three times its standard deviation.

### Experimental setups

**Figure 2.**
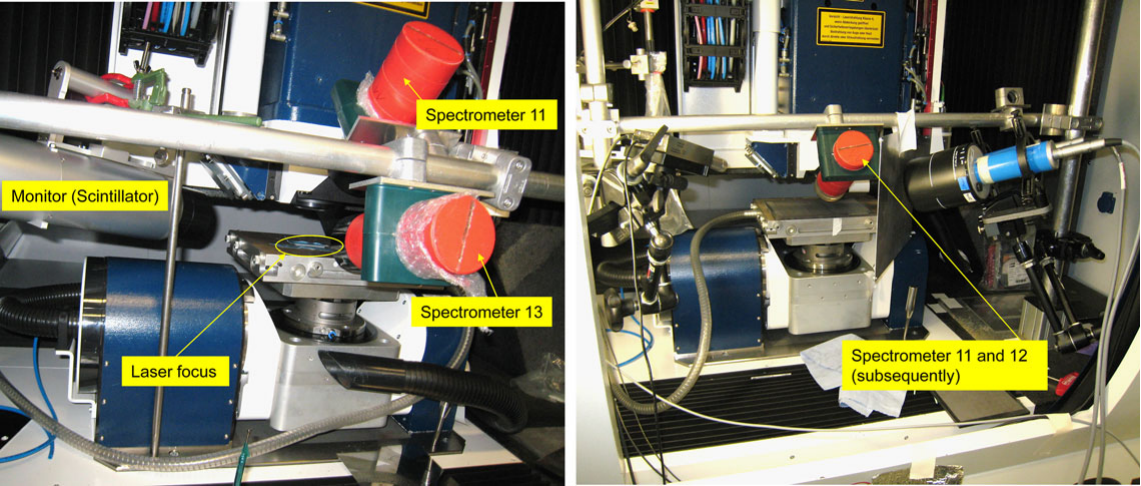
Pictures of the setup during the first (left) and second (right) setup.

Figure 2 shows the measuring setups for two different geometries. Table 2 lists the details of the experiments. The workpiece was fixed on its underside on a vacuum table. For reasons of material availability, the same workpiece surfaces were processed by the laser several times during the irradiations. As a result of material removal, in particular during the first processing, the laser focus probably no longer had its maximum intensity on the workpiece surface during the further processing steps, since the workpiece surface was now somewhat lowered. An additional effect occurs with the Tungsten foil of 0.2 mm thickness, since it was not as flat as the 6 mm metal sheet.

**Table 2.** Details of the experiments.

Parameter	First setup		Second setup	
	Spectrometer 11	Spectrometer 13	Spectrometer 11	Spectrometer 12
Workpiece material	Tungsten		Steel (St37)	Alloy^a^
Workpiece thickness	0.2 mm		6 mm	
Workpiece condition	Plane parallelism and flatness not specified		Plane parallelism and flatness ~15 μm	
Frequency of workpiece processing at the same surface position	One time		Up to seven times	
Angle relative to workpiece surface	46°	13°	31°	
Distance x of the spectrometer front from the laser focus	17 cm		9.76 cm	
Effective irradiation time	2.6 h		3.1 h	2.2 h

^a^92.5% mass fraction tungsten; 3.75% mass fraction iron; 3.75% mass fraction nickel.

For measurement of the background dose in the 30 TLD layers (due to the zero effect in the TLDs and due to natural background radiation), an additional spectrometer was not irradiated.

## DATA EVALUATION AND RESULTS

### Method for the few-channel spectrometer

Following the irradiations, the doses were measured in the 30 TLD layers of each spectrometer and the background dose from the unirradiated spectrometer was subtracted from the doses in the irradiated ones. The dose measurement was calibrated by irradiating separate TLDs to a dose traceable to PTB’s primary standards. This allowed absolute fluence spectra to be determined and absolute dose values in terms of different quantities to be determined therefrom. For evaluation, the doses were related to the effective irradiation time.

In the first 13 TLD layers, doses significantly above background were measured. Only these 13 layers were used for data evaluation, for which a Bayesian method was utilized^(8, 9)^. The main advantages of this method are that pre-information can be included in the evaluation and that, in addition to the results, also their standard uncertainties and coverage intervals are reported. The uncertainty contributions considered are listed in Appendix 2; details will be given in a separate paper^(15)^.

The following pre-information was included in the parametrization, see equation (1). The first term describes the characteristic fluorescence radiation of the target material. Due to the mechanisms of the laser interaction with matter, it is known that the emitted radiation is Maxwellian distributed for electrons and for photons it is dominated by an exponential decrease as the photon energy increases; this is accounted for in the second term. In addition, there were at least ~10 cm of air between the laser focus and the spectrometer front resulting in a significant absorption of low energy radiation, which is represented by the smooth rise via the ‘+cosine’ term in the second line of equation (1). Finally, a drop off to zero fluence was assumed at some unknown particle energy, which is represented by the smooth drop via the ‘−cosine’ term in the fourth line of equation (1).

Significant contributions due to electrons could be ruled out, as discussed in the next subsection. Therefore, only the photon contribution was considered for the subsequent data evaluation. As outlined above, equation (1) was used to parametrize the photon fluence $\Phi {\left(E\right)}_{x}$ at the distance x from the laser focus, i.e. the measuring position (Table 2).

Two additional alternatives utilizing different models compared to equation (1) were also used for the data evaluation in order to identify the most appropriate model. The one represented by equation (1) is the most appropriate, further details will be given in a separate paper^(15)^.

An overall validation of the spectrometer using equation (1) is outlined in Appendix 3.

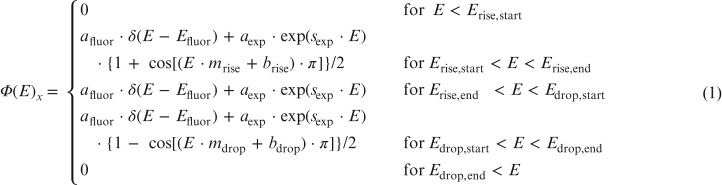

with $a_{\mathrm{fluor}}$ the amplitude of the fluorescence radiation, $E_{\mathrm{fluor}}$ the energy of the fluorescence radiation: 6.5 keV was chosen for the steel target (rounded mean energy of the K_*α*_ and K_*β*_ emission lines from iron) and 9 keV for the tungsten and alloy targets (rounded mean energy of the *L*_*α*_ and *L*_*β*_ emission lines from tungsten), $a_{\exp}$ the amplitude of the exponential decay, $s_{\exp}$ the slope of the exponential decay, $m_{\mathrm{rise}}=1/\left(E_{\mathrm{rise},\mathrm{end}}-E_{\mathrm{rise},\mathrm{start}}\right)$, $b_{\mathrm{rise}}=-m_{\mathrm{rise}}\;\cdot \;E_{\mathrm{rise},\mathrm{end}}$, $m_{\mathrm{drop}}=-1/\left(E_{\mathrm{drop},\mathrm{end}}-E_{\mathrm{drop},\mathrm{start}}\right)$, $b_{\mathrm{drop}}=-m_{\mathrm{drop}}\;\cdot E_{\mathrm{drop},\mathrm{end}}$, $E_{\mathrm{rise},\mathrm{start}}$ the energy where the rise of the spectrum starts, $E_{\mathrm{rise},\mathrm{end}}$ the energy where the spectrum turns into the exponential decay, $E_{\mathrm{drop},\mathrm{start}}$ the energy where the drop of the spectrum starts and $E_{\mathrm{drop},\mathrm{end}}$ the energy where the spectrum drops to zero.

The seven parameters were optimized to match the measured dose values, i.e. to fulfill equation (2) using the program WinBUGS^(16)^. The allowed parameter ranges and their initial start values for the data evaluation are given in Table 3. Due to the absorption in (at least) ~10 cm of air between the laser focus and the spectrometers, only photons with at least 2 keV reach the spectrometers resulting in the parameter range of *E*_rise,start_ starting at 2 keV.

(2) $${\vec{D}}_{\mathrm{meas}}=R\;\cdot \;\vec{\Phi }$$

with the measured doses ${\vec{D}}_{\mathrm{meas}}=\left(\begin{array}{c} D_{1}\\ \vdots \\ D_{N} \end{array}\right)$, in *N* = 30 TLD layers,

**Table 3.** Parameters and their allowed ranges and initial start values for the data evaluation.

Parameter	Range; initial value	Explanation
*a*_fluor_/cm^−2^	0…10^15^; 1 · 10^11^	Amplitude of the fluorescence radiation
*a*_exp_/cm^−2^	0…10^15^; 3 · 10^11^	Amplitude of the spectrum’s exponential decay
*s*_exp_/keV^−1^	−50…50; −0.5	Slope of the spectrum’s exponential decay
*E*_rise,start_/keV	2…10; *2*	Energy at which the spectrum rise starts
*E*_rise,end_/keV	*E*_rise,start_ + 0…20; 3	Energy at which the spectrum rise is finished
*E*_drop,start_/keV	0…500; 250	Energy at which the spectrum drop starts
*E*_drop,end_/keV	*E*_drop,start_ + 0…1000; 500	Energy at which the spectrum ends

the fluences $\vec{\Phi }$ to be determined, $\vec{\Phi }=\left(\begin{array}{c} \Phi_{1}\\ \vdots \\ \Phi_{M} \end{array}\right)$, in *M* = 60 energy channels and

the calculated responses $R=\left(\begin{array}{ccc} R_{1,1} & \cdots & R_{1,M}\\ \vdots & \ddots & \vdots \\ R_{N,1} & \cdots & R_{N,M} \end{array}\right)$.

As the spectrometer positions were different during the experiments (Table 2), the results were related to 10 cm distance from the laser focus. This was performed in two steps: firstly, the quadratic distance law was applied and, secondly, the exponential attenuation law was used to correct for the different air absorptions at the measuring position and at 10 cm distance, see the following equation.

(3) $$\begin{array}{cc} \Phi {\left(E\right)}_{10\;\mathrm{cm}} & \;=\Phi {\left(E\right)}_{x}\cdot {\left\{x/10\;\mathrm{cm}\right\}}^{2}\\ & \cdot \exp\left[-\left\{10\;\mathrm{cm}-x\right\}\;\cdot \;\mu_{\mathrm{en}}\left(E\right)/\rho \right] \end{array}$$

with $\Phi {\left(E\right)}_{10\;\mathrm{cm}}$ the photon fluence at 10 cm distance from the laser focus, $\Phi {\left(E\right)}_{x}$ the photon fluence at the distance x from the laser focus, x the distance of the spectrometer front from the laser focus during the irradiation and $\mu_{\mathrm{en}}\left(E\right)/\rho$ the energy absorption coefficient depending on the photon energy^(17)^.

Note that for *x* larger than 10 cm the correction factor $\exp\left[-\left\{10\;\;\mathrm{cm}-x\right\}\;\cdot \;\mu_{\mathrm{en}}\left(E\right)/\rho \right]$ results in values larger than unity.

### Results: contribution of electron radiation

Preliminary evaluations also took the contribution due to electrons into account in equation (1). For this evaluation, an exponential decay was assumed for photons and a Maxwellian distribution for electrons. The results clearly showed that no significant dose contribution due to electrons was present (of the order of 10^−3^ of the dose due to photons) and, consequently, no significant contribution of electron radiation at all.

### Results: contribution of photon radiation

#### Spectra

**Figure 3.**
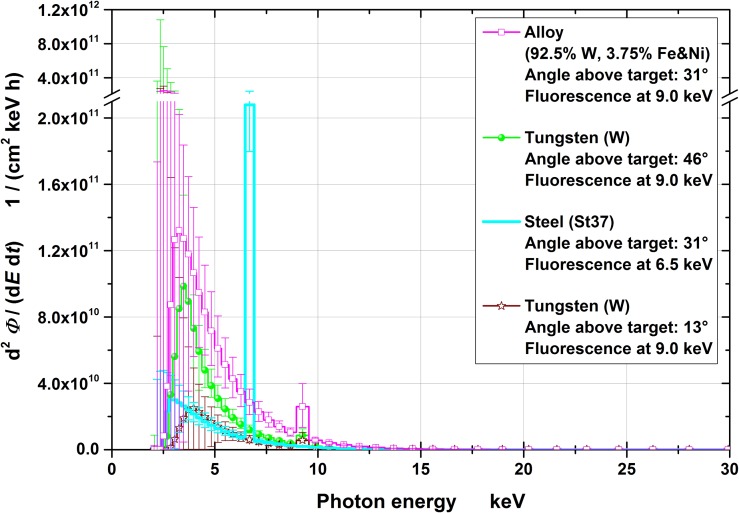
Fluence spectra at the four measuring positions (normalized to the effective irradiation time and 10 cm distance). The uncertainty bars represent the 95% coverage intervals. Note that the ordinate is broken and has a 10 times larger scale after the break.

**Figure 4.**
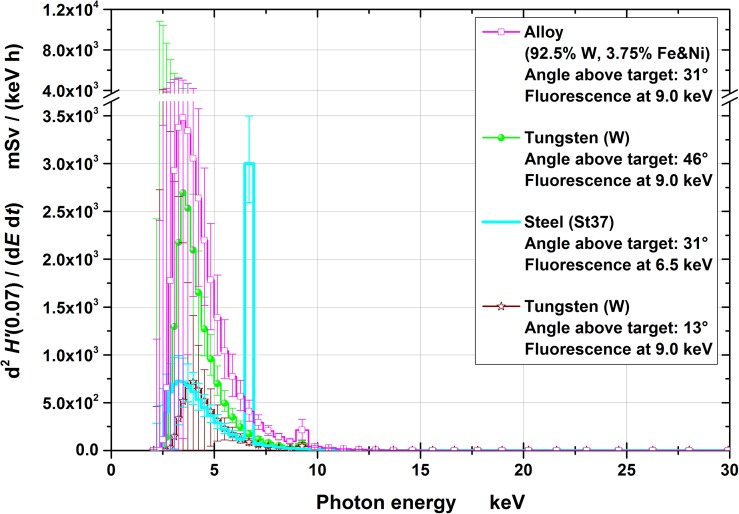
*H*′(0.07) spectra at the four measuring positions (normalized to the effective irradiation time and 10 cm distance). The uncertainty bars represent the 95% coverage intervals. Note that the ordinate is broken and has an eight times larger scale after the break.

**Figure 5.**
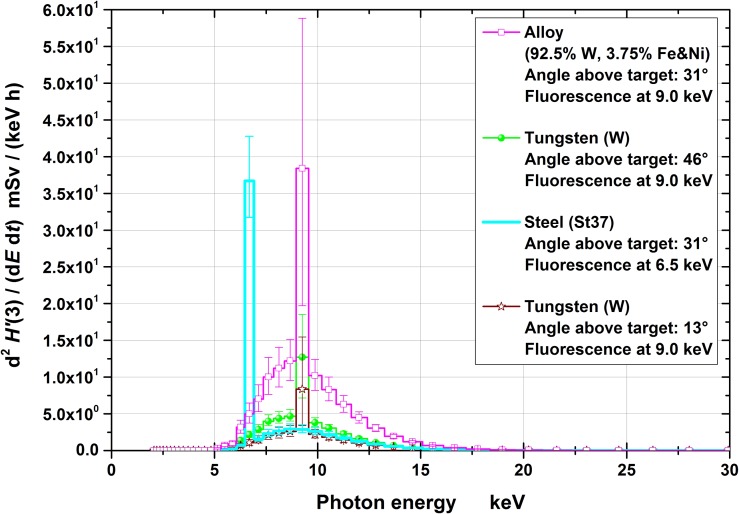
*H*′(3) spectra at the four measuring positions (normalized to the effective irradiation time and 10 cm distance). The uncertainty bars represent the 95% coverage intervals.

**Figure 6.**
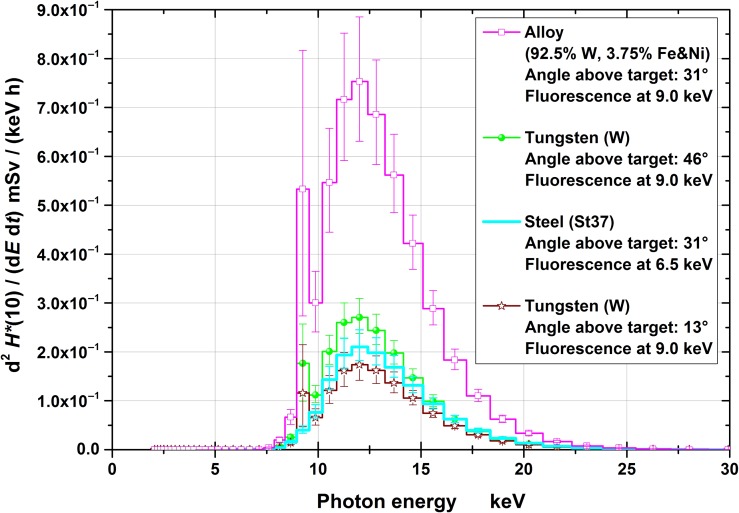
H*(10) spectra at the four measuring positions (normalized to the effective irradiation time and 10 cm distance). The uncertainty bars represent the 95% coverage intervals.

Figures 3–6 show the spectra for the fluence, *Φ*, and the radiation protection quantities *H*′(0.07), *H*′(3) and H*(10), respectively, together with the 95% coverage intervals as uncertainty bars. These intervals represent the region which covers the actual (correct) values (with a probability of 95%). The position of the (correct) value is the less likely, the closer the position is assumed near the edge of the interval.

Conversion of the fluence to the operational quantities was carried out by multiplication with conversion coefficients for mono-energetic photons: for *H*′(0.07) and H*(10) taken from ICRU 57^(18)^ and for *H*′(3) from Behrens^(19)^. The quantities *H*′(0.07), *H*′(3) and H*(10) are relevant for monitoring local skin dose, eye lens dose and effective dose, respectively. From the figures it can clearly be seen that significant contributions exist only to up to ~20–25 keV of photon energy.

The spectra are less uncertain the higher the photon’s energy (Figures 3–6) especially below ~5 keV. This is because the higher the photon’s energy, the deeper the radiation penetrates the spectrometer and, consequently, more TLD layers contain information in their dose values resulting in less uncertain spectral fluences. Photons with energies below 5 keV only penetrate the first TLD layer resulting in practically no spectral information. This is one important reasons for the necessity of taking pre-information into account for the data evaluation.

Although the optimized values of the parameters from equation (1) are not as important as the resulting spectra, it shall be noted that the values for *E*_drop,start_ and *E*_drop,end_ were rather large, i.e. in the order of a few 100 keV. This means that no definite endpoint energy of the spectra was found by the optimization software WinBUGS. This coincides with the assumption that the primary electrons from the plasma are Maxwellian distributed and consequently, the photons follow an exponential decay both with no finite endpoint energy.

#### Dose rate values

By integrating the spectra over the energy range, the total dose rate values were determined (Table 4). The maximum dose rate occurs for the alloy workpiece as a solid sheet, ~4.1 mSv/h for the quantity Ḣ*(10). The corresponding value for the alloy foil is only one-fourth of that. As stated in subsection ‘Experimental setups’, in contrast to the metal sheet the foil was not as flat as the sheet. In addition, the sheet was processed with the laser up to 7 times at the same position. Because of this, the maximum intensity of the laser focus, especially in the case of the foil, was not always directly on the workpiece surface, with the result that the intensity on the actual workpiece surface was lower than indicated in Table 3. As in the case of the spectra, the uncertainties are larger for lower photon energies. This is because the larger the energy, the deeper the radiation penetrates the spectrometer and, consequently, the more TLD layers contain information from that radiation.

**Table 4.** Dose rate values for operational quantities (at 10 cm distance from the laser focus), their standard deviations and 95% coverage intervals.

Workpiece and emittance angle	$\overset{\cdot }{H}$′(0.07) mSv/h	$\overset{\cdot }{H}$′(3) mSv/h	$\overset{\cdot }{H}$*(10) mSv/h
Alloy: 31° above the target	(8.3 ± 2.1) · 10^3^ {±25%}	(76 ± 6) {±8%}	(4.3 ± 0.3) {±7%}
	95%-IV: [4.3;12.5] · 10^3^	95%-IV: [64;89]	95%-IV: [3.8;4.9]
Tungsten: 46° above the target	(4.8 ± 2.6) · 10^3^ {±54%}	(27.7 ± 2.0) {±7%}	(1.53 ± 0.09) {±6%}
	95%-IV: [2.3;12.5] · 10^3^	95%-IV: [24.1;32.0]	95%-IV: [1.36;1.72]
St37: 31° above the target	(3.1 ± 0.4) · 10^3^ {±12%}	(30.5 ± 2.1) {±7%}	(1.21 ± 0.08) {±7%}
	95%-IV: [2.4;3.8] · 10^3^	95%-IV: [26.8;35.1]	95%-IV: [1.06;1.39]
Tungsten: 13° above the target	(1.6 ± 1.4) · 10^3^ {±86%}	(16.8 ± 1.8) {±10%}	(1.04 ± 0.07) {±7%}
	95%-IV: [0.4;5.6] · 10^3^	95%-IV: [13.7;20.6]	95%-IV: [0.90;1.19]

#### Dose contributions from photon energies above 30 keV

The dose contributions from photons with energies above 30 keV were also determined from the spectra (Table 5). The contribution lies below 2·10^−7^, 2·10^−5^ and 4·10^−4^ for the quantities *H*′(0.07), *H*′(3) and H*(10) independent of the workpiece material and emittance angle above the target. Again, the uncertainties decrease for deeper reference depths of the quantity.

**Table 5.** Dose rate contributions above 30 keV photon energy to the operational quantities (at 10 cm distance from the laser focus), their standard deviations and 95% coverage intervals.

Workpiece and emittance angle	$\overset{\cdot }{H}$′(0.07)_>30 keV_/$\overset{\cdot }{H}$′(0.07)	$\overset{\cdot }{H}$′(3)_>30 keV_/$\overset{\cdot }{H}$′(3)	$\overset{\cdot }{H}$*(10)_>30 keV_/$\overset{\cdot }{H}$*(10)
Alloy: 31° above the target	(0.8 ± 0.4) · 10^−7^ {±48%}	(0.88 ± 0.18) · 10^−5^ {±20%}	(1.4 ± 0.3) · 10^−4^ {±21%}
	95%-IV: [0.4;1.9] · 10^−7^	95%-IV: [0.61;1.31] · 10^−5^	95%-IV: [0.99;2.12] · 10^−4^
Tungsten: 46° above the target	(4.1 ± 1.7) · 10^−8^ {±42%}	(7.3 ± 1.2) · 10^−6^ {±16%}	(1.22 ± 0.20) · 10^−4^ {±16%}
	95%-IV: [1.7;8.3] · 10^−8^	95%-IV: [5.1;9.8] · 10^−6^	95%-IV: [0.85;1.61] · 10^−4^
St37: 31° above the target	(1.36 ± 0.29) · 10^−7^ {±22%}	(1.38 ± 0.23) · 10^−5^ {±16%}	(3.2 ± 0.5) · 10^−4^ {±16%}
	95%-IV: [0.89;2.04] · 10^−7^	95%-IV: [0.99;1.88] · 10^−5^	95%-IV: [2.3;4.3] · 10^−4^
Tungsten: 13° above the target	(2 ± 3) · 10^−7^ {±164%}	(1.8 ± 0.5) · 10^−5^ {±31%}	(2.6 ± 0.8) · 10^−4^ {±32%}
	95%-IV: [0;10] · 10^−7^	95%-IV: [1.0;3.1] · 10^−5^	95%-IV: [1.5;4.7] · 10^−4^

#### Mean energies

Finally, the mean energies for the four quantities were determined from the spectra using different types of weighting (Table 6). They range from ~4–6 keV (*Φ*‒ and *H*′(0.07)‒weighted) to up to 13 keV (H*(10)‒weighted) and show no significant tendency with regards to dependence on the workpiece material and/or emittance angle above the target.

**Table 6.** Mean energies of the spectra weighted with fluence and operational quantities (at 10 cm distance from the laser focus), their standard deviations and 95% coverage intervals.

Workpiece and emittance angle	$\bar{E}\left\{\Phi \right\}/\mathrm{keV}$	$\bar{E}\left\{H\prime \left(0.07\right)\right\}/\mathrm{keV}$	$\bar{E}\left\{H\prime \left(3\right)\right\}/\mathrm{keV}$	$\bar{E}\left\{H*\left(10\right)\right\}/\mathrm{keV}$
Alloy: 31° above the target	(5.1 ± 0.5) {±9%}	(4.4 ± 0.3) {±7%}	(9.62 ± 0.03) {±0.4%}	(12.94 ± 0.06) {±0.5%}
	95%-IV: [4.3;6.0]	95%-IV: [4.1;5.3]	95%-IV: [9.55;9.69]	95%-IV: [12.82;13.07]
Tungsten: 46° above the target	(4.7 ± 0.7) {±15%}	(4.3 ± 0.5) {±12%}	(9.53 ± 0.03) {±0.3%}	(12.89 ± 0.05) {±0.4%}
	95%-IV: [3.2;5.8]	95%-IV: [3.3;5.2]	95%-IV: [9.47;9.59]	95%-IV: [12.79;12.99]
St37: 31° above the target	(5.8 ± 0.2) {±4%}	(5.3 ± 0.2) {±4%}	(8.31 ± 0.06) {±0.8%}	(13.44 ± 0.07) {±0.5%}
	95%-IV: [5.3;6.2]	95%-IV: [5.0;5.7]	95%-IV: [8.20;8.44]	95%-IV: [13.31;13.58]
Tungsten: 13° above the target	(5.4 ± 1.0) {±19%}	(4.7 ± 0.8) {±17%}	(9.71 ± 0.06) {±0.6%}	(13.16 ± 0.10) {±0.7%}
	95%-IV: [3.4;7.4]	95%-IV: [3.4;6.6]	95%-IV: [9.60;9.84]	95%-IV: [12.98;13.36]

## CONCLUSIONS

The emission (including its spectral distribution) of laser induced X-rays from materials processing ultra-short pulsed laser system was successfully determined. To achieve this, a TLD-based few-channel spectrometer was mechanically modified to also cover the photon energy region of a few to up to 100 keV. The spectrometer’s response (dose per incident fluence) was newly calculated and the original method of data evaluation (from dose to spectrum) was further developed for this particular application. With this, a reliable method was established to measure pulsed photon spectra in the low energy region up to a 100 keV.

The use of other laser parameters (like focus intensity, wavelength, angle of incidence of the laser beam, etc.), workpieces (both material and surface), and methods used for the materials processing that are different from the ones described in this work will very likely lead to different results for the radiation emission both for photons and electrons.

## Figures and Tables

**Figure C1.**
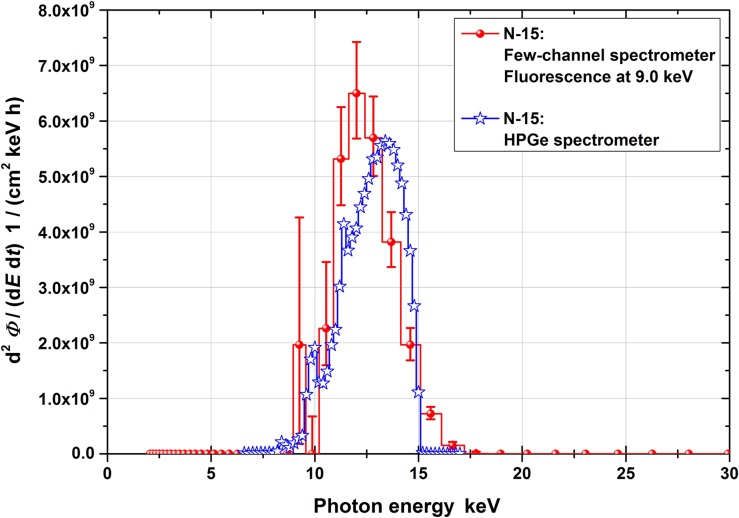
Fluence spectra for the reference radiation quality N-15 (=A-15) from the data evaluation of the spectrometer and, as reference, from a HPGe spectrometer. The uncertainty bars represent the 95% coverage intervals.

**Figure C2.**
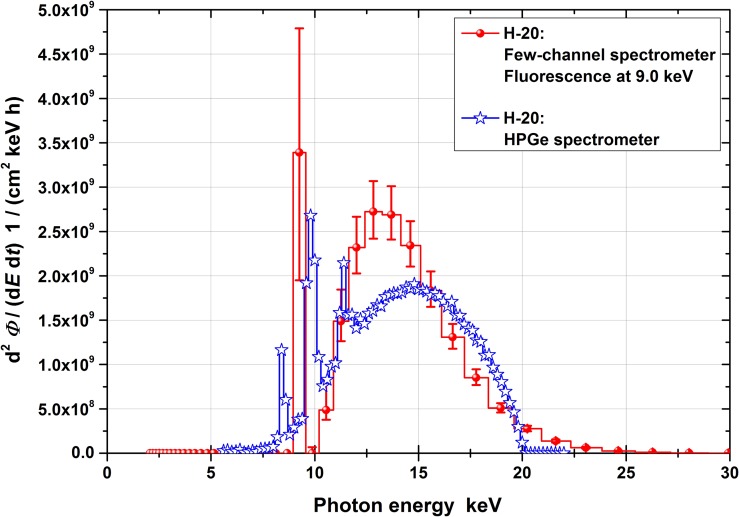
Fluence spectra for the reference radiation quality H-20 (= C-20) from the data evaluation of the spectrometer and, as reference, from a HPGe spectrometer. The uncertainty bars represent the 95% coverage intervals.

**Figure C3.**
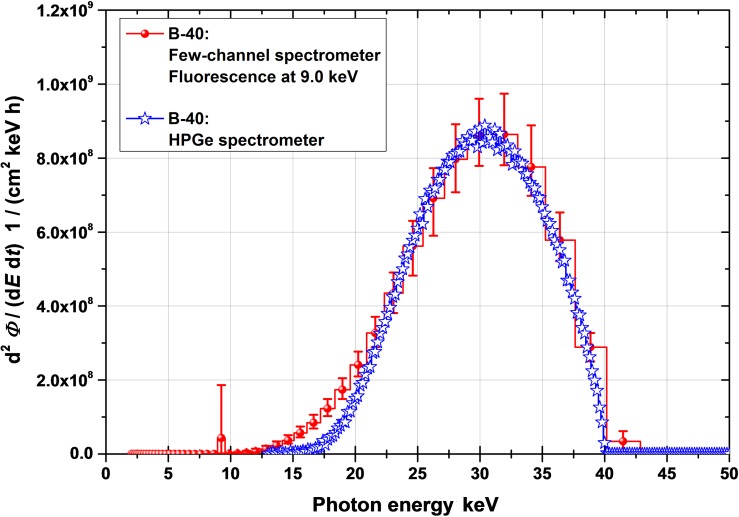
Fluence spectra for the reference radiation quality B-40 from the data evaluation of the spectrometer and, as reference, from a HPGe spectrometer. The uncertainty bars represent the 95% coverage intervals.

**Figure C4.**
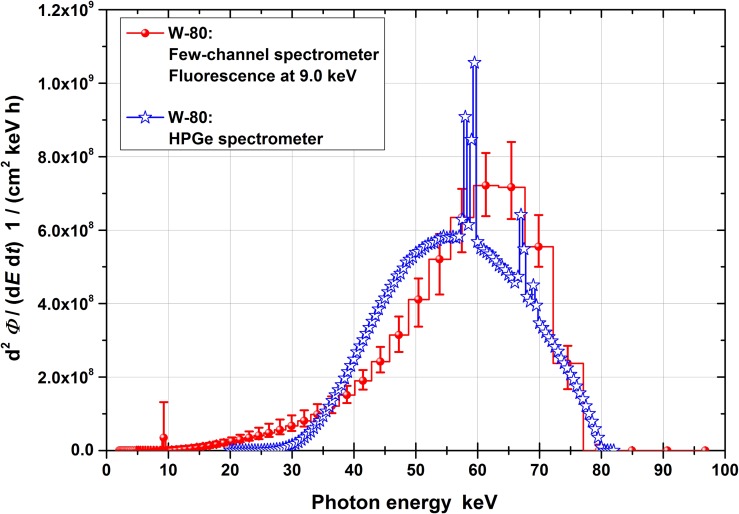
Fluence spectra for the reference radiation quality W-80 (= B-80) from the data evaluation of the spectrometer and, as reference, from a HPGe spectrometer. The uncertainty bars represent the 95% coverage intervals.

## Appendix 1 Details of the few-channel spectrometer

Table A1 lists the filter materials and thicknesses used in the spectrometer. The very first filter made of polycarbonate is doped with graphite so that it is light-tight. This prevents laser light from penetrating the PMMA and reaching the TLDs which are sensitive to light. Figure A1 shows the response functions for photons and electrons. The latter were calculated for 11 cm distance between a point source and the spectrometer front. For measurements performed at other distances the responses are converted via the quadratic distance law to the actual distance during the measurement. Doing so the different depths of the 30 TLD layers within the spectrometer are taken into account.

**Table A1.** Material and thickness of the layers of the few-channel spectrometer.

Filter material	Thickness (g/cm^2^)	No. of TLD layer behind filter
Polycarbonate^a^	0.0147	1
PMMA^b^	0.118	2
PMMA	0.118	3
PMMA	0.118	4
PMMA	0.230	5
PMMA	0.226	6
PMMA	0.490	7
PMMA	0.706	8
PMMA	0.942	9
PMMA	1.18	10
PMMA	0.118	Additional absorber in front of layer 11
Titanium	0.0232	11
Titanium	0.0232	12
Titanium	0.0463	13
Iron	0.0413	14
Iron	0.0413	15
Copper	0.0536	16
Copper	0.0536	17
Copper	0.0893	18
Molybdenum	0.0767	19
Molybdenum	0.0767	20
Molybdenum	0.104	21
Silver	0.110	22
Silver	0.147	23
Silver	0.147	24
Tin	0.183	25
Tin	0.183	26
Tin	0.371	27
Alloy^c^	0.356	28
Alloy	0.356	29
Alloy	0.356	30
Teflon	0.0572	In front of and behind TLD layers 11–30
Teflon spacer	0. 227	Beside TLD layers (layer 11–30)
Lithium fluoride^d^	0.158	Located within Teflon spacers

^a^Doped with graphite.

^b^Chemical composition: C_5_H_8_O_2_; Polymethyl metacrylate (PMMA).

^c^Alloy: 92.5% mass fraction tungsten; 3.75% mass fraction iron; 3.75% mass fraction nickel.

^d^TLD700: LiF (enriched ^7^Li) doped with Mg and Ti.

## Appendix 2 Uncertainty contributions

For the Bayesian data evaluation, the following uncertainty contributions were taken into account during the optimization of the parameters in equation (1):

- the statistical contributions to the dose measurement: in the order of ±2% or larger (type A uncertainties);
- the uncertainty of the absolute dose calibration: ±3% (type B uncertainty);
- the uncertainty contribution due to the distance uncertainty of the spectrometers: ±2.5 and ±5 mm (half width of rectangular distributions) for a distance of 9.76 and 17 cm, respectively, resulting in ~±2 up to 3.5% uncertainty of the dose, depending on the spectrometer’s position and on the position of the TLD layer in the spectrometer (type B uncertainty); and
- the uncertainty of the response matrix’ entries, which was estimated from the uncertainty of the interaction coefficients, the modeling of the spectrometer’s setup (geometry and materials) and the simulation process itself: from ±3 up to ±6% from the upper down to the lower energy end of the response matrix, i.e. from 100 down to 2 keV (type B uncertainty).

Only the statistical uncertainties of the doses (a) are uncorrelated from one TLD layer to the other. The other contributions (b) to (d) are correlated in the following ways: the uncertainty of the absolute dose calibration (b) is 100% correlated among all TLD layers; at the uncertainty due to the distance uncertainty (c), however, the uncertainties in the different TLD layers depend on the layers’ position in the spectrometer; the uncertainties of the response matrix entries (d) are certainly correlated for neighboring energy channels but not for very different energies. These different types of correlation were implemented in the Bayesian data evaluation using the WinBUGS program. Further details will be given in a dedicated publication^(15)^.

Unless otherwise stated, all uncertainties contributions stated above are standard uncertainties and were determined according to the GUM^(21)^.

## Appendix 3 Validation of the overall performance of the spectrometer

To check the overall performance of the few-channel spectrometer, i.e. the determination of the spectrum and the dose in terms of different quantities, it was irradiated in four reference radiation X-ray fields at PTB, N-15 (=A-15), H-20 (=C-20), (B-40) and W-80 (=B-80) (nomenclature according to ISO 4037-1^(22)^; and in brackets according to DIN 6818-1^(23)^), to a dose of *H**(10) = 10.0 mSv, traceable to PTB’s primary standards, at a distance of 2.5 m from the focus of the X-ray tube. The first two X-ray fields were chosen as their spectral range and mean energy is approximately in the energy range of the spectra measured at the laser facilities. N-15 is a strongly filtered X-ray field with an accelerating voltage of 15 kV, H-20 is weakly filtered with a voltage of 20 kV and B-40 and W-80 are medium filtered with voltages of 40 and 80 kV, respectively. The two latter X-ray fields were chosen to complete the energy range up to nearly 100 keV.

Figures C1–C4 show the fluence spectra of the four radiation qualities resulting from the data evaluation of the few-channel spectrometer compared to the spectra measured with a high purity Germanium (HPGe) spectrometer^(24)^. As expected, not all the details of the spectra are reproduced correctly, like the fluorescence radiation where the model for the few-channel spectrometer only uses one energy bin while the HPGe spectrometer resolves, e.g. the α and β lines. However, the energy range, the approximate form of the spectra and their absolute intensity are reproduced quite well. It shall be noted that,

- for the spectrum evaluation, no prior information beyond the ones from equation (1) and Table 3 (both in the main text) was used, i.e. no further energy or dose information at all,
- as the X-ray fields were produced with a Tungsten anode, also 9 keV (for the L-lines) was chosen, not 59 keV (for the K-lines) as the high voltage was assumed to be unknown, and
- apart from the ^137^Cs dose calibration of the TLDs, no normalization was applied to the spectra of the few-channel spectrometer.

The spectra of the HPGe spectrometer were normalized to the irradiated dose of the X-ray fields, *H**(10) = 10.0 mSv by conversion to the operational quantities using the conversion coefficients from fluence to air kerma, *K*_a_/*Φ*, and from air kerma to the operational quantities, *h*′_*K*_(0.07), *h*′_*K*_(3) and *h**_*K*_(10). The mono-energetic conversion coefficients were taken from the literature^(18, 19)^.

Tables C1–C3 show the resulting spectrum characteristics from the data evaluation compared to the ones obtained for the spectra measured with the HPGe spectrometer. The ratios of the values from the few-channel spectrometer and the HPGe spectrometer roughly agree with unity within their 95% coverage intervals. Only the dose contribution due to photons above 30 keV is significantly above zero for H-20 (up 2 · 10^−4^) which it should not be. Further on, for B-40 and W-80 the ratios of the dose contributions above 30 keV significantly deviate from unity, possibly, as no uncertainties are available for the spectra of the HPGe spectrometer^(24)^. Apart from these small deviations, the Bayesian data evaluation for the few-channel spectrometer leads to reliable spectrum characteristics, attributed uncertainties and coverage intervals.

**Table C1.** Dose values from the few-channel spectrometer for operational quantities for the two X-ray reference radiation fields, their standard deviations and 95% coverage intervals, and, in each second line, the ratio to the corresponding values from the reference dosimetry with their 95% coverage intervals.

Reference radiation field; quantity	*H*′(0.07) (mSv)	*H*′(3) (mSv)	*H**(10) (mSv)
N-15			
Dose values: few-channel spectrometer	(111 ± 7) {±6%}	(50.1 ± 2.9) {±6%}	(10.2 ± 0.6) {±6%}
	95%-IV: [99;126]	95%-IV: [45.0;56.4]	95%-IV: [9.1;11.4]
Dose ratios: few-channel spectrometer/HPGe spectrometer	1.16 ± 0.15	1.11 ± 0.13	1.02 ± 0.12
H-20			
Dose values: few-channel spectrometer	(75 ± 4) {±6%}	(36.6 ± 1.9) {±5%}	(11.0 ± 0.6) {±5%}
	95%-IV: [67;84]	95%-IV: [33.2;40.6]	95%-IV: [9.9;12.2]
Dose ratios: few-channel spectrometer/HPGe spectrometer	1.10 ± 0.13	1.13 ± 0.12	1.10 ± 0.11
B-40			
Dose values: few-channel spectrometer	(13.7 ± 0.7) {±5%}	(12.8 ± 0.6) {±5%}	(10.8 ± 0.5) {±5%}
	95%-IV: [12.5;15.1]	95%-IV: [11.7;14.1]	95%-IV: [9.9;11.9]
Dose ratios: few-channel spectrometer/HPGe spectrometer	1.16 ± 0.12	1.12 ± 0.11	1.08 ± 0.11
W-80			
Dose values: few-channel spectrometer	(10.3 ± 0.5) {±5%}	(10.4 ± 0.5) {±5%}	(10.4 ± 0.5) {±5%}
	95%-IV: [9.4;11.3]	95%-IV: [9.5;11.4]	95%-IV: [9.5;11.5]
Dose ratios: few-channel spectrometer/HPGe spectrometer	1.11 ± 0.11	1.07 ± 0.10	1.04 ± 0.10

**Table C2.** Dose contributions above 30 keV photon energy of the spectra from the few-channel spectrometer: for N-15 and H-20, the values, standard deviations and 95% coverage intervals are given while for B-40 and W-80, the ratios to the corresponding values from the HPGe spectrometer with their 95% coverage intervals are stated.

Reference radiation field; quantity			*H*′(0.07)_>30 keV_/*H*′(0.07)	*H*′(3)_>30 keV_/*H*′(3)	*H**(10)_>30 keV_/*H**(10)
N-15	Dose contributions above 30 keV		0	0	0
H-20	Dose contributions above 30 keV		(3.7 ± 0.6) · 10^−5^ {±17%}	(7.7 ± 1.3) · 10^−5^ {±17%}	(2.3 ± 0.4) · 10^−4^ {±17%}
			95%-IV: [2.8;5.2] · 10^−5^	95%-IV: [5.7;10.7] · 10^−5^	95%-IV: [1.8;3.3] · 10^−4^
B-40	Ratios of contrib. above 30 keV:	few-chan. spec./HPGe spec.	1.10 ± 0.06	1.13 ± 0.06	1.16 ± 0.05
W-80	Ratios of contrib. above 30 keV:	few-chan. spec./HPGe spec.	0.91 ± 0.03	0.94 ± 0.02	0.96 ± 0.01

**Table C3.** Ratios of mean energies of the spectra from the few-channel spectrometer and the spectra from the HPGe spectrometer, weighted with fluence and operational quantities. The 95% coverage intervals are given.

Reference radiation field; quantity		$\bar{E}{\left\{F\right\}}_{\mathrm{few}-\mathrm{ch}\;\mathrm{spc}}/\bar{E}{\left\{F\right\}}_{\mathrm{HPGe}.\;\mathrm{spc}}$	$\bar{E}{\left\{H\prime \left(0.07\right)\right\}}_{\mathrm{few}-\mathrm{ch}\;\mathrm{spc}}/\bar{E}{\left\{H\prime \left(0.07\right)\right\}}_{\mathrm{HPGe}.\;\mathrm{spc}}$	$\bar{E}{\left\{H\prime \left(3\right)\right\}}_{\mathrm{few}-\mathrm{ch}\;\mathrm{spc}}/\bar{E}{\left\{H\prime \left(3\right)\right\}}_{\mathrm{HPGe}.\;\mathrm{spc}}$	$\bar{E}{\left\{H⁎\left(10\right)\right\}}_{\mathrm{few}-\mathrm{ch}\;\mathrm{spc}}/\bar{E}{\left\{H⁎\left(10\right)\right\}}_{\mathrm{HPGe}.\;\mathrm{spc}}$
N-15	Energy ratios: few-channel spec./HPGe spec.	0.98 ± 0.01	0.98 ± 0.02	0.98 ± 0.01	0.99 ± 0.01
H-20	Energy ratios: few-channel spec./HPGe spec.	1.00 ± 0.02	1.01 ± 0.03	0.99 ± 0.01	0.99 ± 0.01
B-40	Energy ratios: few-channel spec./HPGe spec.	0.99 ± 0.01	0.96 ± 0.02	0.97 ± 0.01	0.99 ± 0.01
W-80	Energy ratios: few-channel spec./HPGe spec.	1.01 ± 0.01	0.96 ± 0.03	0.99 ± 0.01	1.00 ± 0.01
